# Anatomical and Functional Connectivity at the Dendrodendritic Reciprocal Mitral Cell–Granule Cell Synapse: Impact on Recurrent and Lateral Inhibition

**DOI:** 10.3389/fncir.2022.933201

**Published:** 2022-07-22

**Authors:** S. Sara Aghvami, Yoshiyuki Kubota, Veronica Egger

**Affiliations:** ^1^School of Cognitive Sciences, Institute for Research in Fundamental Sciences (IPM), Tehran, Iran; ^2^Division of Cerebral Circuitry, National Institute for Physiological Sciences (NIPS), Okazaki, Japan; ^3^Neurophysiology, Institute of Zoology, Regensburg University, Regensburg, Germany

**Keywords:** olfactory bulb, recurrent inhibition, lateral inhibition, network model, mitral cell, granule cell, reciprocal synapse, glomerular column

## Abstract

In the vertebrate olfactory bulb, reciprocal dendrodendritic interactions between its principal neurons, the mitral and tufted cells, and inhibitory interneurons in the external plexiform layer mediate both recurrent and lateral inhibition, with the most numerous of these interneurons being granule cells. Here, we used recently established anatomical parameters and functional data on unitary synaptic transmission to simulate the strength of recurrent inhibition of mitral cells specifically from the reciprocal spines of rat olfactory bulb granule cells in a quantitative manner. Our functional data allowed us to derive a unitary synaptic conductance on the order of 0.2 nS. The simulations predicted that somatic voltage deflections by even proximal individual granule cell inputs are below the detection threshold and that attenuation with distance is roughly linear, with a passive length constant of 650 μm. However, since recurrent inhibition in the wake of a mitral cell action potential will originate from hundreds of reciprocal spines, the summated recurrent IPSP will be much larger, even though there will be substantial mutual shunting across the many inputs. Next, we updated and refined a preexisting model of connectivity within the entire rat olfactory bulb, first between pairs of mitral and granule cells, to estimate the likelihood and impact of recurrent inhibition depending on the distance between cells. Moreover, to characterize the substrate of lateral inhibition, we estimated the connectivity *via* granule cells between any two mitral cells or all the mitral cells that belong to a functional glomerular ensemble (i.e., which receive their input from the same glomerulus), again as a function of the distance between mitral cells and/or entire glomerular mitral cell ensembles. Our results predict the extent of the three regimes of anatomical connectivity between glomerular ensembles: high connectivity within a glomerular ensemble and across the first four rings of adjacent glomeruli, substantial connectivity to up to eleven glomeruli away, and negligible connectivity beyond. Finally, in a first attempt to estimate the functional strength of granule-cell mediated lateral inhibition, we combined this anatomical estimate with our above simulation results on attenuation with distance, resulting in slightly narrowed regimes of a functional impact compared to the anatomical connectivity.

## Introduction

**FIGURE 1:**
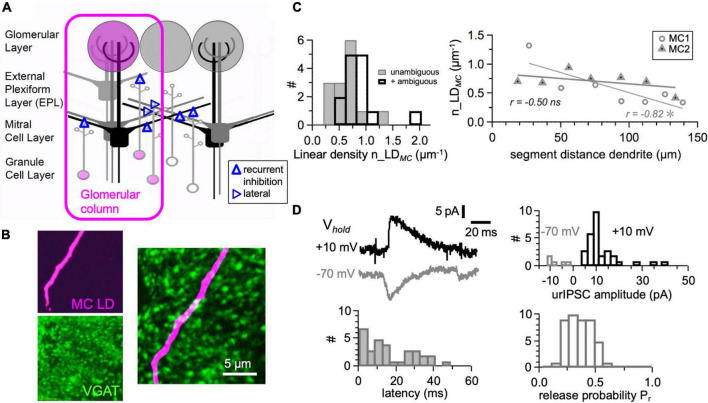
Dendrodendritic interactions between mitral cells and granule cells. **(A)** Scheme of mitral cell–granule cell network. Glomerular column: Ensemble of mitral cells and granule cells that can be excited from a glomerulus. Recurrent inhibition will occur widely, lateral inhibition can happen only *via* the granule cells that are sufficiently excited within the columnar ensemble to generate a global or local spike (if at all, see Section “Discussion”). **(B)** Example double stain of juvenile rat mitral cell lateral dendrite segment and GABAergic presynapses (see Section “Materials and Methods”). Top left: Biocytin label. Bottom left: VGAT-stain. Both z-projections of 5 μm-deep image stack (sectioning at 0.2 μm). Right: Overlay of z-projections within a 1 μm selection of the stack. **(C)** Cumulative histochemical data: Left: Synaptic input density distribution for certain synapses and upper limit (*n* = 14 segments). Right: Density versus distance on the segment. Linear fit (significance of correlation 1-tailed for MC1 *P* = 0.012, MC2 *P* = 0.127, *n* = 7 data points each). **(D)** Unitary-like mitral cell IPSCs are evoked by uncaging of glutamate onto granule cell spines (data from Lage-Rupprecht et al., 2020). Top left: Averaged example traces from two mitral cells at two different holding potentials (−70 mV, +10 mV) and equilibrium potentials E_*Cl*_ (+16 mV, −130 mV). Top right, bottom: Distribution of amplitudes, response latencies, and release probabilities (modified from Figure 1 in Lage-Rupprecht et al., 2020).

The massive presence of inhibitory interneurons at the early processing level is a hallmark of olfactory systems (Shepherd and Greer, 2004). Inhibitory synaptic circuits are thus likely to constitute the core of the central processing unit in early olfactory coding. Since most olfactory receptors detect structural features of odor molecules rather than entire odorants, olfactory coding is synthetic and combinatorial. At the input level of the rodent olfactory bulb, each activated receptor type in turn usually targets two glomeruli and thereby activates their associated sets of principal neurons and interneurons downstream that we will denote as glomerular columns hereafter (Figure 1A). Another specific feature of bulbar circuitry is the large lateral dendritic field span of its principal neurons of up to 2 mm, allowing them to interact with several hundred glomerular columns across the bulb. These long-range dendrodendritic interactions are enabled by quasi-axonal action potential (AP) propagation along the lateral dendrites from which excitation is passed on to inhibitory interneurons, which in turn contact the principal neurons both within and across columns. By now, several subtypes of anaxonic inhibitory interneurons are known to form dendrodendritic reciprocal synapses with the smooth lateral dendrites of the principal mitral cells (MC) and tufted cells (TC): (1) granule cells (GC), with their somata in the GC layer and their apical dendrite extending into the external plexiform layer (EPL) and bearing the reciprocal synapses within large, electrically isolated spines (Price and Powell, 1970b; Woolf et al., 1991; Bywalez et al., 2015) and (2) other types of neurons whose somata are located in the EPL and who feature smooth dendrites. These EPL interneurons consist of various, partially overlapping subpopulations such as parvalbumin neurons, corticotropin-releasing hormone neurons, and somatostatin neurons (e.g., Toida et al., 1994; Hamilton et al., 2005; Kosaka and Kosaka, 2008; Lepousez et al., 2010; Huang et al., 2013); the parvalbumin subtype is by now known to exert substantial inhibition of MCs (Kato et al., 2013; Miyamichi et al., 2013; Liu et al., 2019).

Here, we focused on the role of GCs (1) because GCs are the most abundant neuronal type of the olfactory bulb (in rat > 2⋅10^6^, Richard et al., 2010, versus EPL interneurons <1⋅10^5^, e.g., Parrish-Aungst et al., 2007, in mouse) and also provide the majority of inhibitory inputs onto MCs and TCs compared to parvalbumin interneurons (approximately 90%, Matsuno et al., 2017), (2) because their contribution to inhibition is not well understood (e.g., Fukunaga et al., 2014; Burton, 2017), and (3) because of our long-standing interest in the function of the reciprocal GC spine that has by now allowed us to characterize the operation of the spine microcircuit with regard to its output to MCs. For example, recurrent inhibition by GCs can already be exerted at the single-spine level, since a local glutamatergic MC input can activate the reciprocal microcircuit (Lage-Rupprecht et al., 2020), whereas it is as of yet unknown whether EPL interneuron dendrites can release GABA in response to a single MC AP without further excitation from other inputs.

For similar reasons, we restricted our study to MCs, excluding TC types. There is increasing evidence for a substantial degree of functional and molecular diversity across TCs (e.g., Imamura et al., 2020; Zeppilli et al., 2021); at this point, these subtypes are less well characterized than MCs, especially with regard to their synaptic interactions with GCs (but see Section “Discussion”). Conversely, MC lateral dendrites have been studied both experimentally and in simulations (e.g., Lowe, 2002; Xiong and Chen, 2002; Debarbieux et al., 2003; McTavish et al., 2012; Li and Cleland, 2013; McIntyre and Cleland, 2016). Here, we supplemented these studies with functional data on unitary GC input to MC lateral dendrites (from Lage-Rupprecht et al., 2020) and anatomical data on synaptic density (see Section “Anatomical Basis of Mitral Cell–Granule Cell Dendrodendritic Connectivity”) in order to estimate GC-mediated recurrent inhibition.

A key question in olfactory processing is whether the concept of lateral inhibition as we know it from visual processing can be generalized to the olfactory system. While earlier experimental and modeling results have argued in favor of isotropic contrast enhancement as performed by retinal circuits (e.g., Yokoi et al., 1995; Davison et al., 2003), the discontinuous nature of the olfactory representation itself, where chemotopy may exist only in a limited way (e.g., Soucy et al., 2009), along with observations of sparse and patchy lateral inhibition (e.g., Fantana et al., 2008; Kim et al., 2012; Economo et al., 2016; Lehmann et al., 2016; Shmuel et al., 2019), is sounding a note of caution. However, the used methods are quite diverse in their approaches and both glomerular layer and EPL circuits may well contribute to both isotropic (i.e., radially symmetric) and anisotropic (i.e., patchy) lateral inhibition across glomerular columns. Based on others’ and own functional observations (Arevian et al., 2008; Fukunaga et al., 2014; Bywalez et al., 2015; Burton, 2017; Lage-Rupprecht et al., 2020; Mueller and Egger, 2020), we devised a hypothesis on the role of GCs in olfactory processing, which states that GCs serve to provide lateral inhibition exclusively between coactivated glomerular columns *via* an activity-dependent mechanism located within the reciprocal spines (Lage-Rupprecht et al., 2020; Egger and Kuner, 2021). This hypothesis could reconcile the abovementioned divergent findings on the spatial structure of lateral inhibition. It predicts that the anatomical connectivity between MCs and GCs across glomerular columns should be isotropic in order to allow for maximal flexibility with regard to the functional interaction between coactivated glomerular columns, whereas functional lateral connectivity *via* GCs will be patchy. The prediction of isotropic anatomical connectivity is also one of our key assumptions here. Thus, after first investigating recurrent inhibition, we asked what amount of lateral inhibition could be exerted between any two MCs or the MC ensembles associated with glomerular columns *via* far-reaching dendrodendritic interactions with GCs, based on the anatomical connectivity.

This part of the study builds on an earlier, less elaborate model of anatomical connectivity (Egger and Urban, 2006). Although back then most of the relevant parameters could already be gathered from existing anatomical studies, there were also several less well-defined essential parameters (see below). These gaps have been mostly closed, and thus an update appears timely. Moreover, in the present study, we refined the earlier model by accounting for MC branching and by also covering glomerular columnar ensembles of MCs rather than only single MCs. In addition, as mentioned above, we have now obtained detailed physiological data on synaptic transmission at the reciprocal synapse from GCs to MCs, allowing us to go one step further to estimate the functional impact of both GC-mediated recurrent and lateral inhibition, which so far has been studied in computational network models based on more generic assumptions about synaptic properties (e.g., Davison et al., 2003; Migliore and Shepherd, 2008; McIntyre and Cleland, 2016; Kersen et al., 2022; see Section “Discussion”).

Most important with regard to the level of precision and detail in the description of the anatomical network that governs both recurrent and lateral inhibition are the following recent results:

(1) A crucial anatomical parameter, the linear density of reciprocal synapses along MC lateral dendrites n_LD_*MC*_, was established only recently in a quantitative manner in various studies in mice (Bartel et al., 2015; Sailor et al., 2016; Matsuno et al., 2017), beyond an early seminal study that focused mostly on the ultrastructure and the arrangement of GC spines (Woolf et al., 1991). Because of the importance of this parameter, we also experimentally verified its order of magnitude for juvenile rats (see Section “Anatomical Basis of Mitral Cell–Granule Cell Dendrodendritic Connectivity”), since our functional data were mostly obtained in juvenile rats.
(2) Measurements of the number of MCs per glomerulus N_*MC_GL*_ were significantly improved; recent studies were based on electroporation of single glomeruli in mice converge on N_*MC_GL*_ ≈ 10 (Sosulski et al., 2011; Ke et al., 2013; Liu et al., 2016; Schwarz et al., 2018), while earlier studies estimated larger numbers based on large-scale cell counting (e.g., Allison, 1953; Meisami and Safari, 1981; but see Royet et al., 1989). This parameter is of particular importance for estimating the connectivity between glomerular ensembles of MCs.

## Materials and Methods

### Compilation of Anatomical Parameters

All anatomical parameters in Table 1 are based on the available literature and own data. Whenever possible, data from rat olfactory bulbs were used.

**TABLE 1:** Anatomical parameters (see Section “Compilation of Anatomical Parameters” for further explanation).

#	Parameter	Symbol	Values used in the model	Comments	Source (Species)	Robustness to ±10% change
(1)	Number of MC lateral dendrites	N_LD_*MC*_	5	Average of type I and II MC	Orona et al., 1984 (rat)	
(2)	Total Length of MC lat. dend. per MC	L_LD_*MC*_	12500 μm	Average of type I and II MC	Orona et al., 1984 (rat)	
(3)	Effective total Length of MC lat. dendrites	L_LD_*MC*_eff	10000 μm	Required for model	Mori et al., 1983 (rabbit)	High (±20%) affects only scaling
(4)	Radius of MC dendritic field	R_*MC*_	850 μm	500–1300 μm	Orona et al., 1984 (rat), Mori et al., 1983 (rabbit)	High (∼ ±20%), affects mainly scaling
(5)	Number of branchpoints	N_*BP*_	15	Type I MC	Orona et al., 1984 (rat)	
(6)	Position of branchpoints on MC dendrite (relative to field)	b_1_, b_2_, b_3_	150 μm, 550 μm, 750 μm	see Figure 2A	Mori et al., 1983 (rabbit); with N_LD_*MC*_ and N_*BP*_ matched to obtain L_LD_*MC*_eff	Low (0/-3%; 1/-4%, 2/-5%)
(7)	Average density of synapses on MC lateral dendrite	n_LD_*MC*_	Not used, instead 8	0.64–1.1 μm^–1^	Woolf et al., 1991 (mouse, age 5 weeks), Bartel et al., 2015 (mouse, 2 weeks, 10 weeks), Matsuno et al., 2017 (mouse, 8–10 weeks), Sailor et al., 2016 (mouse, several weeks), own data (rat 2 weeks, see Section “Results”)	High (±20%) affects only scaling
(8)	Effective density on MC lateral dendrite	n_LD_*MC*_eff	1 μm^–1^		Based on ratio dendritic length/field radius (Orona et al., 1984, rat)	
(9)	Total number of GCs that interact with MCs	N_*GC*_	1.5⋅10^6^	2.2⋅10^6^ at 2 weeks, 5⋅10^6^ in adults	Richard et al., 2010 (rat)	Medium (−11%, +8%) affects only scaling
(10)	Number of reciprocal spines per GC	N_*recGC*_	200		Price and Powell, 1970b (rat), Shepherd and Greer, 2004 (rat), Geramita et al., 2016 (mouse)	Does not enter estimate, is used for cross-validation of n_LD_*MC*_eff
(11)	Radius of GC dendritic field	R_*GC*_	50 μm		Orona et al., 1983 (rat), own data	Low (±2%)
(12)	Size of 2D sheet (mid-EPL xy-area)	A_*EPL*_	20⋅10^6^ μm^2^	15–25⋅10^6^ μm^2^	Royet et al., 1989 (rat), Struble et al., 2001 (rat)	Medium (±10%) affects only scaling
(13)	Number of MCs per glomerulus	N_*MC_GL*_	10	Type I and II MC	Liu et al., 2016: M72 mouse: 10 MCs 7 TCs Royet et al., 1989 (rat): 13 MCs Schwarz et al., 2018: mouse M174-9 only 6 MC, 6 d/mTC, 19 sTCs	High (+21/-19%) affects only scaling

*Robustness of connectivity estimate (Figure 5C top): change of parameter value to 110% or 90% of setting.*

#### Comments on Some Parameters

(1, 2) The average number N_LD_*MC*_ and the total length of MC lateral dendrites L_LD_*MC*_ were obtained by averaging the data of Orona et al. (1984) on dendritic lengths for type I and type II individual MCs. Since the relative fractions of MC type I and II cells are not known, we used the arithmetic mean. The delineation between type II MCs and deep or internal TCs seems not entirely clear at this point.
(3) The total effective length of MC dendrites L_LD_*MC*_eff here refers to the projection of MC dendrites into the plane of the EPL; MC dendrites (but not those of TCs) also extend into the vertical direction such that their length extends the horizontal field span of 850 μm by 300 μm (Mori et al., 1983; see Assumption 3 for more details).
(5, 6) The average positions of MC lateral dendrite branch points b_1,2,3_ were estimated based on the large data set by Mori et al. (1983; Figure 4) from rabbits and chosen such that L_LD_*MC*_eff is met for a total number of 5 lateral dendrites and 15 branch points (Orona et al., 1984); there is no similarly detailed study of rat MC branching patterns yet.
(7) Following an early EM study that reconstructed short segments of MC dendrites and the associated GC spines (Woolf et al., 1991), several recent studies analyzed the linear density of inhibitory synapses onto MC lateral dendrites in mice in a quantitative manner (Bartel et al., 2015; Sailor et al., 2016; Matsuno et al., 2017). From the large data set in Sailor et al. (2016), one can gather an inhibitory synapse density of 0.64 ± 0.37 μm^–1^ (*n* = 56 segments with mean length 78 ± 20 μm, average number of puncta per segment 50 ± 29). Bartel et al. (2015) found a mean linear density of 1.1 μm^–1^. Similar values can be derived from Matsuno et al. (2017) for proximal segments (their Figure 9, 0.2 contacts/dendrite area corresponds to roughly 1.2 μm^–1^ for a proximal radius of 1 μm). Interestingly, these authors costained for parvalbumin and found that the total fraction of parvalbumin+ puncta was below 10%. Similarly, Bartel et al. (2015) argued that, because of the large population of GCs compared to other neuron types, most detected contacts are likely to be formed by GCs. Our own measurements in rat MCs complement these data (see Sections “Measurement of Inhibitory Synapse Density on Rat Mitral Cell Lateral Dendrites n_LD_*MC*_” and “Anatomical Basis of Mitral Cell–Granule Cell Dendrodendritic Connectivity”). It is under debate whether inhibitory contacts are clustered or rather homogenously distributed; in any case, sections devoid of any GC synapses are probably shorter than 10 μm (Bartel et al., 2015, their Figure 5). Lacking more precise data, we decided to assume a uniform density along the dendrites as the first approximation (see Assumption 2).
(8) We accounted for the reduced effective length of MC dendrites [see comment on (3) above and Assumption 3] by increasing our result for the density n_LD_*MC*_ to an effective density n_LD_*MC*_eff = 1 μm^–1^.
(9) Because of the restriction of our approach to the GC-MC subnetwork, we reduced the literature value for the total number of GCs by the number of cells that might be part of TC subnetworks (see Assumption 1 for more details).
(10) While the number of reciprocal spines per GC N_*recGC*_ is not relevant for our connectivity estimate (see below, Eq. 6), it is useful to validate the above parameter choices (see Assumption 1).
(11) The lateral extent of the GC dendritic field was measured from the reconstructed GCs shown in Orona et al. (1983) (*n* = 40 cells) and from a set of GCs filled with a fluorescent dye and imaged with two-photon microscopy (Egger et al., 2003, 2005; *n* = 29 cells). The two groups had a virtually identical mean horizontal dendritic field radius of R_*GC*_ = 50 ± 40 μm (S.D.).

### Measurement of Inhibitory Synapse Density on Rat Mitral Cell Lateral Dendrites n_LD_*MC*_

Inhibitory synapse density on rat MC lateral dendrites was measured based on colocalization of a cytoplasmic dendritic stain and VGAT-puncta (Panzanelli et al., 2007) in a set of *n* = 14 dendritic segments from 2 MC proximal lateral dendrites. MCs from acute brain slices (300 μm thickness) of juvenile rats (P14; see Chatterjee et al., 2016 for ethics statement and brain slice preparation, since the slices used here were prepared for that study) were filled with Biocytin (0.5 mg/500 μl internal solution) for 10 min *via* the recording pipette during whole-cell recordings (pipette resistance < 5 MΩ). Slices were fixed (4% paraformaldehyde, 0.2% picric acid, and 0.1% glutaraldehyde in 0.1 M PB) and kept at least overnight. Slices were embedded in agar and resectioned (50 μm thickness). After washing, slices were incubated in 1% sodium borohydrate in PBS for 30 min, washed, and then incubated in primary antiserum overnight (anti-VGAT, developed in rabbit, dilution 1:5000, # A-2052, SIGMA, Taufkirchen, Germany). After washing, slices were incubated in secondary antiserum overnight (Alexa 488-anti rabbit IgG, dilution 1:200, #A-11008, Invitrogen, Waltham, MA, United States) and finally incubated with Alexa 594-streptavidin (1:2000, Invitrogen) for 90 min. Dual-channel Z-stacks (sectioning 0.2 μm) were taken on a confocal microscope (Olympus Fluoview 300, Hamburg, Germany) and analyzed manually by Fluoview software within the first top 5 μm of the slices. Contacts were counted as unambiguous if there was a clear overlap between puncta and dendrite in at least two of the three projection planes (xy, xz, and yz) and as ambiguous if there was an overlap only within one of the three projections. In an earlier study from our laboratory, contacts established by light microscopical techniques were verified by subsequent electron microscopy, yielding a correct hit rate of approximately 80% (Karube et al., 2004).

### Simulations

**FIGURE 2:**
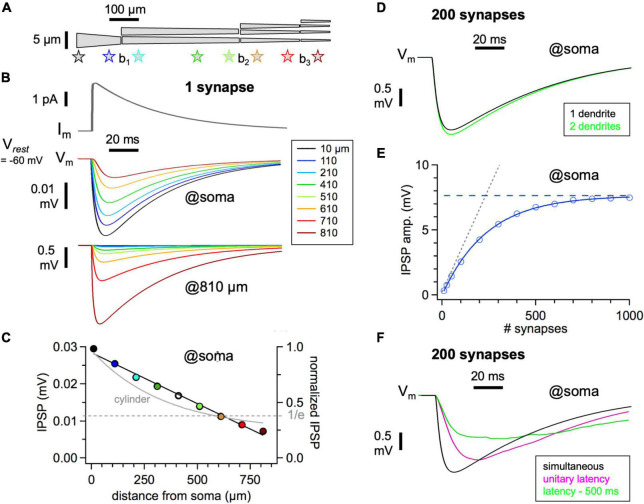
Simulation of granule cell-mediated recurrent inhibition. **(A)** Model anatomy of one mitral cell lateral dendrite. Branchpoints at b_1_ = 150 μm, b_2_ = 550 μm, b_3_ = 750 μm. Total length 850 μm, the total number of branchpoints 3. Tapering: first segment 4–2.5 μm, second segment 2.5–2 μm, third segment 2–1 μm, fourth segment 1–0.5 μm. **(B)** Top: Simulation of IPSC recorded at the soma under physiological conditions (10 μm from soma, g_*syn*_ = 200 pS, V_*m*_ = −60 mV, E_*Cl*_ = −80 mV). Middle: Simulation of IPSP generated at the same location and at various distances, as recorded at the soma. Bottom: IPSP at the same locations as in panel **C** but recorded at the distal site (810 μm). **(C)** Cumulative somatopetal attenuation along the dendrite, absolute (left axis) and normalized to the extrapolated amplitude at 0 μm (normalized IPSP, right axis). Gray line: Simulation in a cylindrical neurite without tapering. **(D)** Somatic recurrent inhibition exerted by 200 synapses distributed equally across 1 lateral dendrite of the mitral cell model (black trace) and with the proximal 80% of synapses shifted to another lateral dendrite (green trace). The difference between the traces indicates the reduced shunting of the distal 20% of synapses in the second case. **(E)** Recurrent summated IPSP amplitude at the soma versus a number of (equidistant) synapses on one dendrite. Dotted line: linear summation of unitary IPSP. Dashed line: saturation due to shunting and reduction in driving force. **(F)** Black trace: same simulation as in panel **(D)**. Magenta trace: same, but with onset latencies distributed according to Figure 1D. Green trace: the temporal extent of latency values further expanded, to 500 ms.

We used a compartmental cable model of an MC to simulate the inhibitory synaptic inputs onto it. The morphology of the dendritic tree was adopted from Orona et al. (1984); one primary dendrite and five similar lateral dendrites tapered non-linearly from 4 to 0.5 μm along the trunks and branches, based on the study of Mori et al. (1983). Figure 2A shows the lateral dendrite geometry (see also Table 1). The total extent of the dendrite matches the radius of the MC dendritic field R_*MC*_ = 850 μm within the xy-plane.

The model was implemented in NEURON 7.4 along with Python (Hines et al., 2009) for both a passive and an active dendritic tree. In both cases, the axial resistance is set to be 100 Ωcm and the membrane conductance is 2e^–4^ S/cm^2^. These passive parameters were adjusted to match the measured unitary IPSCs proximal to the soma (Lage-Rupprecht et al., 2020), considering both the amplitude and time course of the IPSC (Figure 2B). The unitary synaptic inhibition is obtained by mimicking a single square pulse of 1 mM GABA release for 3 ms (as in our previous simulations of glutamate release, e.g., Bywalez et al., 2015) and by implementing GABA receptor kinetics according to Destexhe (1998), with a maximum conductance of 200 pS (based on our functional data, see Section “Results”) and a reversal potential E_*Cl*_ = −80 mV (or E_*Cl*_ = −70 mV in a subset of simulations). We did not implement a gradient in synaptic conductance along the lateral dendrite, since the available experimental evidence is ambiguous (Lowe, 2002). The random onset latency of the inhibitory synapses used in Figure 2F was generated from a Gamma distribution function fitted to the experimental data presented in Figure 1D.

### Implementation of the Anatomical Connectivity Model

All routines were implemented in IGOR (Wavemetrics, OR, United States). Integrations were performed numerically. We evaluated the robustness of the connectivity function (Eq. 9) to parameter variations of ±10%. The rightmost column of Table 1 shows the resulting sensitivities in terms of the average percentage change in the connectivity. Connectivity model assumptions aside from the parameter choices in Table 1 are described in the Section “Model for Recurrent Mitral Cell–Granule Cell Connectivity and Lateral Mitral Cell–Granule Cell–Mitral Cell Connectivity.”

## Results

### Anatomical Basis of Mitral Cell–Granule Cell Dendrodendritic Connectivity

Apart from a seminal early EM study that was restricted to shorter segments of MC lateral dendrites (total summed length 63 μm, Woolf et al., 1991), the density of inhibitory contacts along lateral MC dendrites n_LD_*MC*_ was established only recently (see Section “Introduction”). Since this synapse density is a crucial parameter for the efficiency of both recurrent and lateral inhibition and all the published studies were done in mice, we performed a qualitative test in juvenile rats (inhibitory synapses labeled with Anti-VGAT and lateral dendrite with Biocytin, see Section “Materials and Methods”; Figures 1B,C) and obtained a lower limit of 0.65 ± 0.26 μm^–1^ (*n* = 14 lateral dendrite segments from *n* = 2 MCs, counting only unambiguous puncta; upper limit including ambiguous puncta: 0.83 ± 0.33 μm^–1^; mean segment length 19.2 ± 4.7 μm, total analyzed length 269 μm). This result is in rather a close accordance with the mouse immunohistochemistry data, especially with the study of Sailor et al. (2016). Across segments, linear density was fairly homogenous with the exception of the first segment in MC 1 (Figure 1C).

For the two-dimensional connectivity model described further below, we used an increased effective synaptic density n_LD_*MC*_eff = 1 μm^–1^ that accounts for the fact that MC dendrites are not running in a planar fashion (Mori et al., 1983; Orona et al., 1984; see also Table 1).

### Functional Inhibitory Impact of Individual Granule Cell Spine Inputs

Our previous study of single GC spine output triggered by local two-photon uncaging of glutamate yielded a set of IPSCs from proximal inhibitory inputs (<50 μm from the soma, Lage-Rupprecht et al., 2020). Since these IPSCs are caused by the vesicular release of GABA upon local excitation of the reciprocal spine, we considered them to reflect true unitary inputs. At a holding potential V_*hold*_ = +10 mV and a chloride Nernst potential E_*Cl*_ = −130 mV, these inputs had an average amplitude of 12 ± 8 pA (*n* = 32, mean ± *SD*, example in Figure 1D). This amplitude was increased by a factor of two because of antagonistic interactions of the uncaging compound DNI-Glu with GABAergic currents (block of spontaneous IPSC amplitudes by 50%, Lage-Rupprecht et al., 2020), resulting in a synaptic conductance g_*syn*_ = I_*syn*_/(V_*hold*_ − E_*Cl*_) = 170 ± 120 pS. Using this conductance to predict the IPSC amplitude under different conditions (E_*Cl*_ = +16 mV, V_*hold*_ = −70 mV, used in another subset of experiments) yields −15 pA, or −7.5 pA in DNI. This value is very close to the measured mean value of −8 ± 4 pA in these experiments (in DNI, *n* = 5, Figure 1D), further validating g_*syn*_.

Thus, under more physiological conditions (V_*m*_ = −60 mV, E_*Cl*_ = −80 mV), we obtained I_*syn*_ = 3.4 ± 2.4 pA. For lack of more precise data, we assumed that the conductance does not depend on its distance to the soma (Lowe, 2002). We also used a less hyperpolarized E_*Cl*_ = −70 mV in a second set of simulations, following the argument by McIntyre and Cleland (2016) that this choice better reflects the physiological situation in adult animals *in vivo*, where then I_*syn*_ = 1.7 pA.

### Simulation of Recurrent Inhibition

For the simulation of recurrent inhibition, we used a compartmental model (see Section “Materials and Methods”), with the dendritic morphology of an MC lateral dendrite as shown in Figure 2A. Figure 2B shows a single IPSC originating from a synapse at a distance of 10 μm from the soma, based on the unitary conductance established above. Passive parameters and kinetics of GABA release were adjusted to match our recorded IPSC kinetics and also previously established kinetics in the current clamp condition (based on spontaneous IPSPs recorded in MCs at −60 mV, rise time 12 ± 7 ms, half duration 40 ± 15 ms, *n* = 27 IPSPs in 9 MCs, data set from Egger et al. (2005); these IPSPs might originate from different sources than GCs but the passive parameters should be similar, see Section “Discussion”). Unitary IPSPs were simulated originating from synapses at the indicated distances from the soma. Our simulations (Figure 2B) thus predict a unitary IPSP amplitude on the order of 0.03 mV for proximal input and below 0.01 mV for inputs beyond 700 μm from the soma. Such small amplitudes are below the detection limit in conventional, non-averaged whole-cell current-clamp recordings (see Section “Discussion”).

**FIGURE 3:**
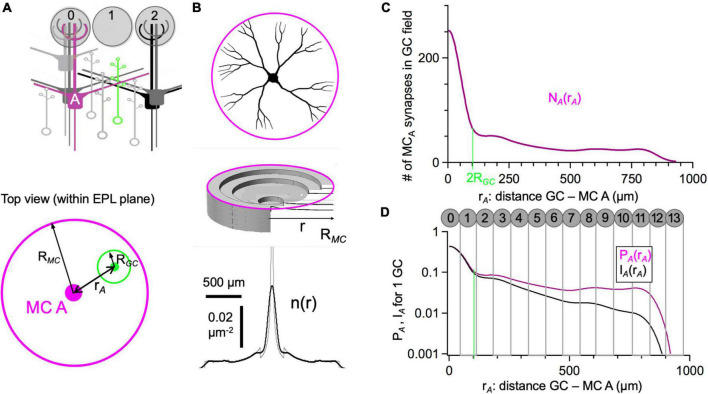
Anatomical connectivity between individual mitral cells and granule cells. **(A)** Scheme of the MC-GC network, below the top view of the dendritic fields of MC A (magenta, field radius R_*MC*_) and a GC (green, field radius R_*GC*_). r_*A*_: distance MC-GC. **(B)** Synaptic density for single mitral cell with branching secondary dendrites as described in Eq. 1. The top graph shows a schematic view of a branching neuron, and the middle graph illustrates how the synaptic density was calculated with the position of the steps corresponding to the average branchpoint position. The bottom graph shows the average radial synaptic density n(r) for a branching cell including smoothing of the branchpoint positions. The gray line corresponds to the unsmoothed density as given in Eq. 1. **(C)** Number of synapses of MC A that a given GC at position r_*A*_ can access (Eq. 3). 2R_*GC*_ indicates the distance at which there is no more overlap with the GC dendritic field with the peak of n(r). **(D)** Probability P_*A*_ that a GC at position r_*A*_ will indeed connect to MC A (Eq. 6) and effective inhibitory impact I_*A*_ (Eq. 7, from Figure 2C). The spacing of glomeruli is indicated (diameter of glomeruli scaled to fit all 4,000 glomeruli into A_*EPL*_, see Section “Results”).

**FIGURE 4:**
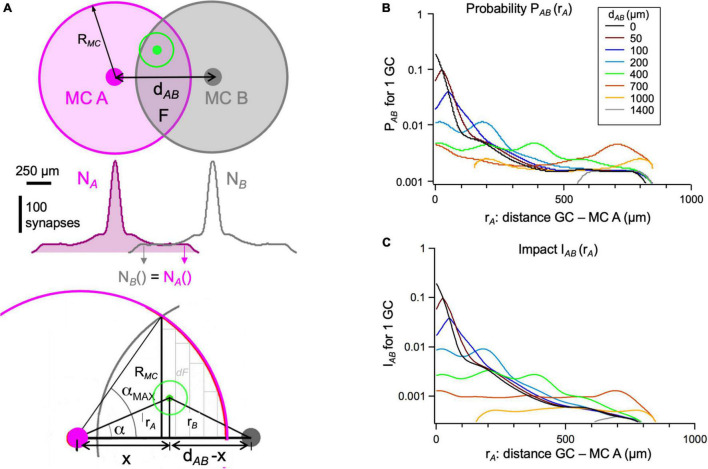
Anatomical connectivity between pairs of mitral cells *via* one granule cell. **(A)** Top: Top view of the dendritic fields of MC A (magenta), a second MC B (gray) at distance d_*AB*_, and a GC (green) within the overlap area F of the MCs’ dendritic fields. Middle: Illustration of symmetry in N_*A*_(r) and N_*B*_(r). Bottom: Geometrical parameters relevant for the calculus in Eqs 9, 10 (see also Table 2). r_*A*,_ r_*B*_: distance of GC from MC A resp. B. α: angle between the position of GC soma r_*A*_ and d_*AB*_. α_*MAX*_: maximally possible α for the respective d_*AB*_. x: distance of projection of GC position onto d_*AB*_ from MC A; relevant for integration. The gray rectangles represent the integration increments *dF*. **(B)** Probability P_*AB*_ of a GC at position r_*A*_ to connect to both A and B (Eq. 6), plotted for a set of distances d_*AB*_ between mitral cells A and B (0–1400 μm). The righthand peak in the curves for 200–700 μm is due to the increased synaptic density around the soma of MC B. Parameters as given in Table 1. **(C)** I_*AB*_: correction of P_*AB*_ for a distance-dependent reduction in IPSP impact because of attenuation (Figure 2C, Eq. 7).

**FIGURE 5:**
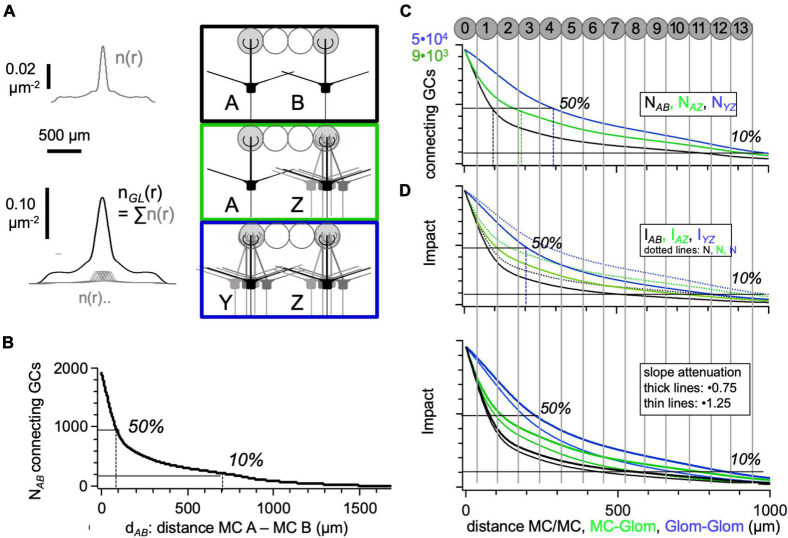
Number of granule cells providing connectivity between two mitral cells or glomerular mitral cell ensemble. **(A)** Synaptic density distribution for single MC n(r) as in Figure 3B and for a glomerular ensemble of mitral cells n_*GL*_(r) (see Section “Materials and Methods”). **(B)** Number of GCs N_*AB*_ that interconnects a pair of MCs versus their distance d_*AB*_ (Eq. 9). The gray bars illustrate the average dimensions of glomeruli within the network (2⋅r_*GL*_ = 120 μm). **(C)** Number of GCs that interconnect a pair of glomerular MC ensembles (blue line) or a single MC and an ensemble (green line), compared to the MC-MC case (black line). For better comparison, all three connectivity functions are scaled to the same size at d_*AB*_ = 0, with the respective maximal numbers indicated in the respective color. **(D)** Top: Number of GCs as above but corrected for their functional impact. Dotted lines: Functions from above for comparison. Arbitrary units on the y-scale. Bottom: Impact with varying degrees of attenuation. The slope of the linear attenuation (fit in Figure 2C) is increased or decreased by 25%. The spacing of glomeruli is indicated (diameter of glomeruli scaled to fit all 4000 glomeruli into A_*EPL*_, see Section “Results”).

Figure 2C shows that the attenuation with distance is almost linear, a result of the implemented dendrite tapering (not of branching, not shown). The somatopetal space constant is on the order of 650 μm, close to the space constant used in a detailed earlier simulation of inhibition in MC lateral dendrites (McIntyre and Cleland, 2016, their lambda 850 μm in a dendrite with a diameter of 2 μm). Note that our MC anatomy is scaled down by a factor of 0.75 to account for the projection into the EPL/xy-plane (see Section “Compilation of Anatomical Parameters”); thus, in a real geometry, attenuation of distal inputs will be accordingly stronger because of the longer extent of the dendrite. Importantly, the result on attenuation is used further below to estimate the recurrent and lateral inhibitory impact of GCs based on our anatomical connectivity models, including a robustness test for modified degrees of attenuation (Figures 3D, 4C, 5C). Finally, synapses situated on the distal dendrite cause a much larger simulated local voltage deflection due to the higher input resistance (Figure 2B bottom).

**TABLE 2:** Additional important variables and their symbols.

Variable	Symbol
Number of reciprocal synapses per MC	N_*synMC*_
Density of granule cells on EPL sheet	n_*GC*_
Synaptic density of single MC (radial coordinates)	n(r)
Synaptic density of glomerular MC ensemble (radial coordinates)	n_*GL*_(r)
Distance of GC from MC A, B, respectively	r_*A*_, r_*B*_
Distance between MCs A, B	d_*AB*_
Coordinate of projection of r_*A*_ onto d_*AB*_	x
Number of synapses of MC A and B within the dendritic field of GC	N_*A*_, N_*B*_
Probability of connection between a GC and MC A or B	P_*A*_, P_*B*_
Inhibitory impact of GC on MC A	I_*A*_
Probability of connection between MC A and B *via* GC	P_*AB*_
Number of interconnecting GCs between MC A/glomerulus Y and MC B/glomerulus Z	N_*AB*_, N_*AZ*_, N_*YZ*_
Angle between the position of GC soma relative of MC A and shortest distance d_*AB*_ between MC somata	α
Area of overlap between dendritic fields of MC A and B	F
Inhibitory impact of MC A/glomerulus Y on MC B/glomerulus Z	I_*AB*_, I_*AZ*_, I_*YZ*_

Next, we investigated the summation of multiple simultaneous IPSPs at the soma, with active synapses distributed with equal spacing along the dendrite. Figure 2D shows the result for 200 co-active synapses, and Figure 2E the amplitude of inhibition versus the number of coactive synapses, indicating a saturation at ∼1000 coactive synapses that lies at −67.5 mV, well below the maximally possible IPSP amplitude of 20 mV (at E_*Cl*_ = −80 mV). This limitation is due both to a decrease in the driving force, characteristic of purely passive dendritic integration (e.g., Tran-Van-Minh et al., 2015), and to increased shunting of distal inputs by proximal inputs, as can be illustrated by simulations with different numbers and distributions of synapses (Vida et al., 2006; David et al., 2008; Figures 2D,E).

In order to approximate the strength of GC-mediated inhibition to be expected in the wake of an MC AP that propagates into the lateral dendrites, we chose the fraction of activated synapses in accordance with our finding for the probability of unitary reciprocal GABA release, which factors in the probability of release of glutamate from the MC dendrite (P_*r*_ = 0.5, Egger et al., 2005) yielding P_*rec*_ = 0.15 (Lage-Rupprecht et al., 2020; Figure 1D), and distributed the onset of release according to the measured latency distribution (also Figure 1D), which was smoothed before random assignment of values to individual synapses. The resulting recurrent IPSP was accordingly reduced in amplitude and broadened (Figure 2F). Asynchronicity of GABA release is likely to be yet more expanded, since our experiments did not account for potentially delayed release from MCs, and time constants on the order of 500 ms were obtained in classical dendrodendritic inhibition experiments. Further broadening will ensue, as shown also in Figure 2F.

### Model for Recurrent Mitral Cell–Granule Cell Connectivity and Lateral Mitral Cell–Granule Cell–Mitral Cell Connectivity

As first described in Egger and Urban (2006), we reduced the connectivity problem within the external plexiform layer (EPL) to the connectivity on a two-dimensional sheet, projecting all reciprocal synapses onto this area, i.e., the mean surface area of the EPL. The first task was to determine the recurrent connectivity between a given MC-GC pair. Next, the estimate of lateral connectivity corresponded to the number of GCs within the overlap area of the dendritic fields of two MCs A and B that are connected to both. The connectivities between two glomerular ensembles, i.e., between all the MCs that are part of a glomerular columnar ensemble or between a single MC and its own or another glomerular ensemble, can be calculated *via* the same approach. Aside from the parameter settings listed in Table 1, the connectivity model is based on the following five assumptions:

#### Assumption 1: The Density of Mitral Cell and Granule Cell Synapses Is Homogenous in the External Plexiform Layer

The density of granule cells n_*GC*_ can be estimated by projecting the total number of GCs onto the mean surface area of the external plexiform layer (EPL).


(1) n≈GCN/GCAEPL

The size of this sheet is roughly given by the EPL volume (6.6 mm^3^, e.g., Royet et al., 1989; Struble et al., 2001) divided by the EPL depth (300–400 μm, e.g., Orona et al., 1984): A_*EPL*_ ≈ 20 mm^2^ (extent in mid-EPL).

However, not all bulbar GCs are available for MC connectivity. While there might be GCs that connect to both MCs and TCs (Arnson and Strowbridge, 2017, identity of interneuron subtype unclear), there is also the notion of anatomically segregated subnetworks (Mori et al., 1983; Orona et al., 1983), which has been reinforced by functional data indicating different connectivities and roles of TCs versus MCs in odor processing (e.g., Ezeh et al., 1993; Nagayama et al., 2004; Fukunaga et al., 2012; Geramita and Urban, 2017). Based on the total dendritic lengths (12000 μm MCs versus 5000 μm TCs, Orona et al., 1984), the rather similar linear synapse densities on both MCs and TCs (Sailor et al., 2016), and the somewhat lower numbers of TCs versus MCs per glomerulus (e.g., Liu et al., 2016; this is the least well-defined number), the number of synapses provided by MCs alone is roughly 70% of the total number of MC and TC synapses, and therefore the number of possibly connecting GCs is reduced from the total number N_*GC*_ by the same fraction to 1.5⋅10^6^ GCs. Thus, for the MC-GC network modeled here, we used n_*GC*_ ≈ 1.5⋅10^6^/20 mm^2^ = 0.075 μm^–2^.

The validity of these settings can be checked by comparing the total number of synapses in the projected EPL area from the GC and the MC points of view:

##### Granule Cell View

N_*GC*_⋅N_*recGC*_ = 1.5⋅10^6^ GCs⋅200 spines per GC yields a total number of GC spine output synapses of 300⋅10^6^.

##### Mitral Cell View

Synapses on lateral dendrites per glomerular ensemble: N_*MC_GL*_⋅L_LD_*MC*_eff⋅n_LD_*MC*_eff = 10⋅10^4^⋅1 = 10^5^, then, the total number of MC synapses (4000 glomeruli in rat, Royet et al., 1989) is 400⋅10^6^. This synapse number also contains synapses with other interneurons, the fraction of which is estimated to be at least ∼10% (Matsuno et al., 2017).

Hence, the total GC and MC synapse numbers are roughly consistent, also validating our setting of n_LD_*MC*_eff = 1 μm^–1^.

#### Assumption 2: Synapses Are Evenly Distributed Along the Lateral Dendrites of Mitral Cells

While anatomical and functional data indicate both short-range and long-range inhomogeneities in synaptic distribution (Price and Powell, 1970a; Mori, 1987; Woolf et al., 1991; Lowe, 2002; Bartel et al., 2015), these data do not allow for a more precise description: other recent measurements did not find any systematic variation (Sailor et al., 2016; see also Section “Materials and Methods”) and our own qualitative data in rat did also not show high fluctuations across the analyzed segments (Figure 1D). A decrease in linear density n_LD_*MC*_ with distance from the soma might be expected because of the tapering of lateral dendrites, but according to Bartel et al. (2015), this effect is not very strong, because the surface density of contacts is actually increasing with smaller dendritic diameters (see e.g., their Figure 5F). Future evidence for systematic changes in linear density with distance could easily be incorporated into the density function (Eq. 2 below).

#### Assumption 3: The Synaptic Density Profile of Mitral Cells and of Glomerular Ensembles of Mitral Cells Is Radially Symmetric

This approach represents the average MC synaptic distribution, neglecting the position of individual lateral dendrites (see also Davison et al., 2003). The average MC dendritic fields are radially symmetric (Price and Powell, 1970a; Mori et al., 1983; Orona et al., 1984). More importantly, labeling of glomerular MC ensembles *via* electroporation has confirmed that a glomerular ensemble’s lateral dendrite distribution is by and large symmetric (Sosulski et al., 2011; Ke et al., 2013; Liu et al., 2016; Schwarz et al., 2018).

Since MC lateral dendrites are not running straight in parallel with the EPL orientation (in contrast to TC lateral dendrites, Mori et al., 1983; Orona et al., 1984) but instead wiggle in the vertical dimension such that the total length of a dendrite (without branches) surpasses its actual field span of 850 by 300 μm or ∼35%, we accounted for this property by decreasing the effective lengths of lateral dendrites (with regard to the projection into the EPL plane) and at the same time increasing the synaptic density on the lateral dendrite n_LD_*MC*_ accordingly, such that the effective density n_LD_*MC*_eff is 1 μm^–1^ instead of 0.65 μm^–1^ (see also Section “Materials and Methods,” Table 1). We distributed the total number of synapses per MC N_*synMC*_ = L_LD_*MC*_⋅n_LD_*MC*_ = L_LD_*MC*_eff⋅n_LD_*MC*_eff = 10000 dropping off with 1/r on a disk with radius R_*MC*_. The branching of the lateral dendrites was taken into account by adding three rings of synaptic densities that also dropped off with 1/r, with an inner radius at the average position of branch points b_1_, b_2_, b_3_, and with outer radius R_*MC*_ (Figures 3A,B; Eq. 1). In our model, there was just one additional branchpoint per lateral dendrite for every further ring (Figure 2A), yielding a total number of 15 branchpoints (in line with type I MCs in Orona et al., 1984).


(2) $$\begin{array}{cc} & n\,\left(r\right)=\sum_{i=0}n_{i}\,\left(r\right)\\ & n_{i}\,\left(r\right)=0\,\mathrm{for}\,r<b_{i}\\ & n_{i}\,\left(r\right)=n_{0}\,\left(r\right)\,\mathrm{for}\,b_{i}<r<R_{\mathrm{MC}}\\ \text{with} & n_{0}\,\left(r\right)=\frac{N_{o}}{R_{\mathrm{MC}}}\cdot \frac{1}{2\,\pi \,r}\\ \text{and} & N_{o}\,\mathrm{such}\,\mathrm{that}\,\int_{0}^{R_{\mathrm{MC}}}n\,\left(r\right)\cdot 2\,\pi \,r\,\mathrm{dr}\\ & =N_{\mathrm{synMC}} \end{array}$$

The synaptic density needs to satisfy the condition that its integral is equal to the total number of dendrodendritic synapses per MC; the factor N_0_ must be chosen accordingly. The soma and apical dendrite also bear reciprocal synapses with GCs, albeit possibly at a reduced density in comparison to the lateral dendrites (Price and Powell, 1970a; see also Benson et al., 1984; Naritsuka et al., 2009). To account for these synapses and to avoid singularities, we used a constant density value around the MC soma that corresponds to the synaptic density n(r) at 10 μm. Finally, n(r) was smoothed with a Gaussian function with width σ = 40 μm (Figure 3B).

To assemble an ensemble of MC lateral dendrites belonging to the same glomerular column n_*GL*_(r), we distributed the N_*MC_GL*_ = 10 MCs per glomerulus according to the distribution of “sister mitral cells” (Buonviso et al., 1991), with a mean average distance of ∼50 μm and a total radius of 200 μm (9 cells placed at distances ±10, ±30, ±50, −70, +90, −110 μm from a first central cell, Figure 5A). Similar distributions have also been observed in more recent research (Kikuta et al., 2013; Schwarz et al., 2018).

#### Assumption 4: The Granule Cells-Mediated Anatomical Connectivity Between Individual Glomerular Ensembles Is Spatially Isotropic

While detailed anatomical data on this issue are not available as of yet (since their quantification would require large-scale electron microscopy and reconstruction), currently available evidence indicates that dedicated dendrodendritic connections between certain glomeruli seem unlikely to play a major role in MC-GC connectivity: MCs and yet more so glomerular MC ensembles have radially symmetric dendritic fields as described above, probably to maximize the spatial range of the glomerular input/output. Using a multi-electrode stimulation device, we also consistently observed that a given GC can be excited (and even fired) from several adjacent glomeruli both in rats and mice (Stroh et al., 2012; Chatterjee et al., 2016; Lukas et al., 2018), in line with results based on optogenetic activation (Burton and Urban, 2015). Thus, a given GC is likely to belong to more than one glomerular column, again arguing against specific connectivities.

Yet, this assumption may be a major simplification; for example, structural and synaptic plasticity (Chatterjee et al., 2016; Huang et al., 2016; Sailor et al., 2016) may result in specifically enhanced connectivity between glomerular ensembles belonging to relevant odor representations (e.g., behaviorally relevant odors that were chosen for a discrimination task or odors of a newly discovered type of food source in the wild). In any case, as outlined in the introduction, isotropic anatomical connectivity would allow for maximal flexibility with regard to the binding of co-active columns.

#### Assumption 5: There Is Just One Reciprocal Synaptic Contact per Coupled Granule Cell–Mitral Cell Pair

This assumption relies both on the previous assumption (no preferred targets) and morphological evidence: Since a given GC dendritic branch was so far not found to contact the same MC branch two times (Woolf et al., 1991; see also Pressler and Strowbridge, 2017) and because of the geometric arrangement of cells, with GC dendrites oriented perpendicularly to the MC dendrites, it appears unlikely that a given GC will contact the same MC more than one time, except for GCs very close to the MC soma. This assumption is further supported by our estimate (Figure 3D): the probability for one contact P_1_ between any GC-MC pair that is within reach of each other is fairly low (on the order of <0.1 for GCs whose dendritic fields do not overlap with the MC soma where the density of MC synapses is highest). The probability for two contacts between the same MC-GC pair, P_2_, is then in first approximation equal to (P_1_)^2^, i.e., on the order of <10^–2^.

However, if we applied this stochastic view to the contacts between a GC and a glomerular ensemble of MCs, the probability that a proximal GC will contact the ensemble more than one time would be rather high and would need to be corrected for if the number of connected GCs is of interest. In particular, if a GC was situated right below the glomerulus, P_1_10_ was close to 1; thus, in this case, the chance for multiple contacts was also very high. We accounted for the possibility of multiple contacts between a GC and a glomerular ensemble of MCs by a binomial correction [subtracting the probabilities for double and triple contacts from the probability for at least one contact by the same cell, which is 1 − (1 − P_1_)^10^, with the probability for no contact at all being (1 − P_1_)^10^].

### Connectivity Between a Mitral Cell and a Granule Cell Depending on Their Distance

To estimate the connectivity between two mitral cells A and B, we first calculated the number of synapses of MC A that a GC at a certain position r_*A*_ within the dendritic field of cell A can “see” in its own dendritic field D, N_*A*_. This function can be approximated rather well by integrating the density of mitral cell synapses n_*A*_(r) over the radius of a GC’s dendritic arbor, at the known distance between the GC and the mitral cell A, r_*A*_, which itself is in the first approximation


(3) $$N_{A}\,\left(r_{A}\right)=\int n_{A}\,\left(r\right)\,\mathrm{dD}\approx n_{A}\,\left(r_{A}\right)\cdot \pi \cdot R_{\mathrm{GC}}^{2}$$

We did this for all possible GC positions, which yielded the function N_*A*_(r_*A*_) as shown in Figure 3D. Now N_*B*_(r) can be calculated from N_*A*_(r) using the geometric relationship between r_*A*_ and r_*B*_ shown in Figure 4A, which depends on the distance between A and B, d_*AB*_, and the angle α of the GC position relative to d_*AB*_.


(4) $${\text{N}}_{B}\,\left({\text{r}}_{A}\right)={\text{N}}_{A}\,\left({\text{r}}_{B}\right)$$

(5) $${\text{r}}_{B}=\sqrt{{\text{d}}_{A\,B}^{2}-2\,{\text{d}}_{A\,B}\,\text{x}+{\text{x}}^{2}\cdot \left(1+\tan^{2}\alpha \right)}$$

The probability that the GC at position r_*A*_ is connected to MC A, P_*A*_(r_*A*_), is the number of available MC synapses N_*A*_ times the number of the GC’s spines (i.e., the chances for the GC to make a contact) divided by the number of all the GC spines of all GCs within the dendritic field D (i.e., the partner synapses of all GCs available for A). Equation 6 is a first approximation that relies on our Assumption 5, i.e. that there is just one reciprocal synaptic contact per coupled GC-MC pair. Note that the number of reciprocal spines per GC N_*recGC*_ was canceled.


(6) $$\begin{array}{c} {\text{P}}_{A}=\frac{{\text{N}}_{A}\cdot {\text{N}}_{r\,e\,c\,G\,C}}{{\text{N}}_{r\,e\,c\,G\,C}\cdot {\text{n}}_{G\,C}\cdot \pi \cdot {\text{r}}_{G\,C}^{2}}\Rightarrow \\ {\text{P}}_{A}\,\left({\text{r}}_{A}\right)=\frac{{\text{N}}_{A}\,\left({\text{r}}_{A}\right)}{{\text{n}}_{G\,C}\cdot \pi \cdot {\text{r}}_{G\,C}^{2}} \end{array}$$

P_*A*_ is shown in Figure 3D, along with the functional inhibitory impact I_*A*_ (Williams and Stuart, 2003) of the GC at position r_*A*_ on the somatic voltage of MC A. Because of the attenuation of the IPSP with distance, factored in as the normalized IPSP nIPSP(r) (Figure 2C), inhibition originating from more distal GCs is reduced accordingly:


(7) I(r)AA=P(r)AA⋅nIPSP(r)A

P_*A*_ and I_*A*_ can be related to the arrangement of glomeruli above, showing that there is high connectivity/impact within the first to the second surrounding ring of glomeruli and a rather constant connectivity and decaying impact further out. The glomeruli were scaled in order to fit onto A_*EPL*_ ≈ 20 mm^2^ (4000 glomeruli in rat ⇒ area per glomerulus ≈ 5000 μm^2^, resulting in an effective glomerular diameter of ≈ 70 μm).

### Connectivity Between Two Mitral Cells and/or Glomerular Ensembles of Mitral Cells *via* Granule Cells Depending on Their Distance

The probability that a certain GC at position r_*A*_ in the overlap area F will be connected to both A and B is the product of the individual probabilities for connections to A and B (Figure 4B):


(8) $${\text{P}}_{A\,B}\,\left({\text{r}}_{A}\right)={\text{P}}_{A}\,\left({\text{r}}_{A}\right)\cdot P_{B}\,\left(r_{A}\right)\,\overset{\mathrm{Eq}\,. 4}{=}{\text{P}}_{A}\,\left({\text{r}}_{A}\right)\cdot {\text{P}}_{A}\,\left({\text{r}}_{B}\right)$$

The inhibitory impact I_*AB*_ of such an MC-GC-MC connection can be estimated by replacing either P_*A*_ or P_*B*_ with its impact (Eq. 7), since the spreading IPSP within the lateral dendrite of the receiving MC is attenuated, whereas the conduction of the action potential along the lateral dendrite of the excited MC is not much affected by attenuation (e.g., Lowe, 2002; Xiong and Chen, 2002; Djurisic et al., 2004; Figure 4C).

Finally, to obtain the number of all GCs in F that will be connected to both A and B, we integrated the product of P_*AB*_ and the density of GCs within the overlap area F.


(9) $$\begin{array}{ccc} {\text{N}}_{A\,B}\,\left({\text{d}}_{A\,B}\right) & = & \int {\text{n}}_{G\,C}\cdot {\text{P}}_{A\,B}\,\left(r,\alpha \right)\,\text{dF}\\ & = & 2\cdot {\text{n}}_{G\,C}\,\int_{d_{A\,B}/2}^{{\text{R}}_{M\,C}}\bar{{\text{P}}_{A\,B}\,\left(\text{r}\right)}\,\frac{\text{dF}}{\text{dr}}\,\text{dr}\\ \text{F}\,\left(\text{r}\right) & = & {\text{r}}_{M\,C}^{2}\,\text{arccos}\,\left(\frac{\text{r}}{{\text{R}}_{M\,C}}\right)-\text{r}\,\sqrt{{\text{R}}_{M\,C}^{2}-{\text{r}}^{2}}\Rightarrow \text{dF}\\ & = & -2\,\sqrt{{\text{r}}_{M\,C}^{2}-{\text{r}}^{2}\,\text{dr}} \end{array}$$

Since P_*AB*_ (r) was not fixed within an increment of the integration dF (see Figure 4A) but depended on the angle α, its mean value within dF should to be calculated beforehand:


(10) $$\begin{array}{ccc} \bar{{\text{P}}_{A\,B}\,\left(\text{r}\right)} & = & \frac{\int_{0}^{\alpha \,\_\,\max}{\text{P}}_{A\,B}\,\left(r,\alpha \right)\,\text{dα}}{\alpha \,\_\,\max}\\ {\text{P}}_{A\,B}\,\left(r,\alpha \right) & = & \frac{{\text{N}}_{A}\,\left({\text{r}}_{A}\right)\cdot {\text{N}}_{A}\,\left({\text{r}}_{B}\right)}{{\text{n}}_{G\,C}^{2}\cdot \pi^{2}\cdot {\text{R}}_{G\,C}^{4}}\\ & & \text{with}\,{\text{r}}_{A}=\frac{\text{r}}{\text{cos}\,\alpha },{\text{r}}_{B}\,\text{from}\,\text{Eq}\,. 4\\ \alpha \,\_\,\max & = & \arccos\left(\frac{\text{r}}{{\text{R}}_{M\,C}}\right) \end{array}$$

The result for the set of parameters in Table 1 is shown in Figures 5B,C. The relation to the glomerular map above is indicated by vertical lines, with glomeruli sized as in Figure 3D. There are three regimes: Connectivity is the highest for connections between MCs within the same home glomerulus 0 and/or the first ring of adjacent glomeruli and then drops off rather quickly, with another decline at the border of the regime where the MC somata are farther apart than the effective dendritic field span, d_*AB*_ > R_*MC*_ = 850 μm. This second drop is explained by the lost overlap between the proximal somatic regions with their high synaptic density and the lateral dendrites of the other MC, respectively.

Next, we calculated the connectivities for either single MCs and a glomerular MC ensemble or between two entire glomerular ensembles. Glomerular MC ensembles are better captured by our mean-field approach than individual MCs since the synaptic density of a glomerular ensemble of MC lateral dendrites would be smoother and better resemble the assumed isotropic density n(r) (Assumption 3). However, a correction for multiple contacts between a given GC and the MC ensemble should be introduced (Assumption 5). To calculate the number of GCs that connect 2 glomerular ensembles Y and Z within the MC-GC network, we assembled the synaptic density of a glomerular ensemble from a set of N_*MC_GL*_ = 10 MCs based on the reported distributions of sister MCs, which were substantially broader than a glomerular diameter, on the order of 200 μm (Buonviso et al., 1991; Ke et al., 2013; Kikuta et al., 2013; Schwarz et al., 2018; Assumption 3). Therefore, the synaptic density distribution n_*GL*_(r) was considerably broadened in comparison to the density n(r) of a single MC (Figure 5A).

While the resulting numbers of connecting GCs (Figure 5C top left) may appear high at first glance, they are not implausible. If, for example, we looked at the maximal number of GCs that could provide intraglomerular inhibition across the MCs within a glomerular ensemble (≈50.000 GCs), these would constitute roughly 25% of the entire set of GCs within the dendritic field of the glomerulus‘ MCs (n_*GC*_⋅π⋅(R_*MC*_ + 100 μm)^2^≈ 210.000 GCs). Figure 5C also shows the connectivity between a single MC and an MC glomerular ensemble.

We found that, for glomerular MC ensembles instead of single MCs, the observed connectivity regimes were substantially broadened. Now the steep initial drop to 50% was extending well into the fourth ring of glomeruli. The switch to the intermediate regime was far less pronounced than for the single MC case. This intermediate region ranged from the fourth ring to at least the eleventh, where connectivity might still be sufficient to excite GCs *via* all the MCs that belong to MC A’s glomerular ensemble and thus could potentially mediate lateral inhibition (but see Section “Discussion”).

However, this broadening is reduced if we took into account the reduced inhibitory impact of more distant GCs due to the attenuation of IPSPs, as shown in Figure 5D. As stated above, the attenuation was accounted for by multiplying P_*AB*_, i.e., the integrand in Eq. 10, with the normalized distance-dependence of the IPSP, nIPSP(r) (from Figure 2D). Now the regime of high impact between two MC ensembles was narrowed to three rings of glomeruli and the impact beyond these three rings was also substantially reduced in comparison to the purely anatomical model.

### Robustness of Results

Our anatomical estimate is fairly robust with respect to parameter variations (Table 1). Only changes in parameters that determine the areas of integration (R_*MC*_, R_*GC*_) also change the shape of the connectivity function. All other parameter changes solely affect its scaling.

As to the combination of the anatomical connectivity estimate with our results from compartmental modeling, we also tested for variations in the degree of attenuation/effective space constant, since this parameter is not well established experimentally (see Section “Discussion”) and influences the inhibitory impact by more distal synapses. Figure 5D (bottom) shows the result of modifying the linear slope of the normalized attenuation from Figure 2D by a factor of 0.75 (shallower) and 1.25 (steeper). The second modification can be interpreted to account for the reduced dendritic length in our model since a synapse that is located at 800 μm in the model is located at 1100 μm in the real morphology. These modifications affected the inhibitory impact across interconnected glomeruli more substantially than the impact between single MCs, but overall, their effect was rather weak.

## Discussion

Ever since the 1980s, detailed quantitative anatomical studies of the vertebrate olfactory system have contributed to our knowledge of the underpinnings of neural networks in the olfactory bulb, and by now, many parameters that contribute to GC-MC connectivity are well characterized. Yet, while our general approach is based on substantial experimental evidence, both anatomical and functional, there are still many assumptions on insufficiently well-known parameters, so our results are to be interpreted with a grain of salt and should certainly not be taken as precise predictions.

### Functional Implications for Inhibition Originating From Granule Cell Reciprocal Spines

One lesser-known property is electrotonic conduction within MC lateral dendrites. So far, investigations of passive spread have been mostly restricted to the MC apical dendrite. Lowe (2002) characterized lateral dendritic inhibitory inputs using flash photolysis of GABA with high spatial resolution at distances up to 150 μm and obtained somewhat ambiguous results. Investigation of more distal inputs, direct dendritic recordings, or voltage-sensitive dye imaging of passive conduction in lateral dendrites has not been performed yet. While it is by now safe to assume that active conduction in lateral dendrites works almost as well as in the apical dendrite, the passive properties, in particular the passive space constant, remain poorly characterized. Still, in line with earlier studies (McIntyre and Cleland, 2016), our simulations predict a substantial attenuation of distal IPSPs.

Our simulations based on experimental data show that even for proximal inputs the size of a unitary IPSP originating from a GC spine is <0.05 mV, i.e., below the noise threshold at the MC soma (e.g., noise σ = 0.11 mV at V_*rest*_ = −60 mV, Diba et al., 2004), and thus is unlikely to exert any influence on MC spiking at all. However, in the case of recurrent inhibition, it has to be considered that there will be the release of GABA from ∼ 200 GC spine inputs per lateral dendrite: there are up to 10,000 reciprocal spines per MC of which up to 15% will release GABA, according to the experimentally established reciprocal release probability P_*r*_ (Figure 1D). This population response will summate (Figure 2) and thereby can substantially hyperpolarize the MC soma. Still, this GC-mediated hyperpolarization is unlikely to prevent MC spiking (e.g., Fukunaga et al., 2014) because it cannot prevent the glomerular MC spike (Chen et al., 2002) from depolarizing the MC soma beyond the threshold of voltage-gated sodium channels. Nevertheless, the summated IPSP can delay spiking and thus possibly infer synchronization of MCs, as also pointed out earlier (e.g., Schoppa, 2006; McTavish et al., 2012; McIntyre and Cleland, 2016). Such synchronization in the gamma-beta range in turn is likely to promote the binding of odor representations across the olfactory bulb and/or to enable transmission of stimulus features other than identity to the piriform cortex (e.g., Kashiwadani et al., 1999; Doucette et al., 2011; Dalal and Haddad, 2022).

GCs are known to also establish dendrosomatic contacts with MCs (Price and Powell, 1970a; Benson et al., 1984; Naritsuka et al., 2009; Pressler and Strowbridge, 2017). For type S GCs, it has been observed that these contacts are housed in spines much larger than the usual gemmule (Naritsuka et al., 2009), thus the functional impact of these synapses might also be more substantial than what we predicted here. Thus, the detectable spontaneous IPSPs recorded from MCs elsewhere and also by us (e.g., Desmaisons et al., 1999; Egger et al., 2005) might originate either from EPL interneurons, dendrosomatic (GC) inputs and/or synchronous release of GABA from several GCs.

Summation/integration of inhibitory inputs has received little attention so far but should follow similar rules as the passive integration of excitatory inputs. We predict substantial shunting effects to occur during recurrent inhibition, resulting in a saturation of the amplitude with an increasing number of activated synapses that happens well below the limitation by the decreasing driving force and thus is related to shunting. The effect of shunting on MC AP timing and its ensuing ability to mediate synchronization of MCs has been explored in a previous simulation (David et al., 2008).

### Anatomical Connectivity

Our connectivity model is based on a statistical mean-field approach that projects all synaptic contacts into two dimensions and provides an analytical solution for averaged morphologies (Egger and Urban, 2006). While this approach might overly simplify interactions between individual MCs, it is likely to cover interactions between and within glomerular ensembles of MCs fairly well. Other recent studies used three-dimensional approaches (Migliore et al., 2014; Kersen et al., 2022) with realistic MC morphologies, also restricted to the MC-GC subnetwork, to generate reduced OB network models. Because of the reduced numbers of cells and different connectivity rules, their network connectivity measures are difficult to compare with our results, which are based on the inclusion of the entire MC-GC OB network. The Kersen et al. (2022) model found that, for a pair of MCs, the number of shared GCs and lateral inhibition (in terms of a reduction of MC firing rate) dropped off less rapidly with distance, reaching 50% at 200–300 μm distance (rather than at 100 μm), but then there was no intermediate regime of connectivity/impact. As to experimentally established connectivities, pairwise MC-GC connections have been notoriously difficult to investigate, even though the detection of MTC inputs activated by glomerular stimulation is unproblematic (e.g., Schoppa and Westbrook, 1999; Egger et al., 2005, etc.). In a heroic slice study, MC-GC connectivity of GCs positioned vertically below MCs was found to be well below the values predicted by our model (Pressler and Strowbridge, 2017, 3–5% compared to our >10%; 4% in Kato et al., 2013). Several factors might contribute here: MCs are mostly contacted by deep GCs which are more likely to be damaged during slicing, as well as part of the lateral dendrites of any MC. Moreover, our mean-field approach might systematically overestimate connectivity, at least between single cells.

### Anatomical Versus Functional Lateral Connectivity

Specific GC-mediated lateral connectivity (rather than isotropic) might exist and be subject to learning (e.g., Fantana et al., 2008; Huang et al., 2016); in any case, the isotropic substrate used here as a first approximation serves to facilitate such experience-dependent changes, which may affect not so much the anatomical network but the functional lateral connectivity emerging from it, which underlies several additional influences. First, electrotonic attenuation within the lateral MC dendrites will strongly reduce inputs from the more distal parts of the lateral dendrite, even though the space constant of these dendrites is rather large. We included this attenuation in our estimate of inhibitory impact. Second, the propagation of APs in MC lateral dendrites is very likely dynamically regulated: later APs in a burst might propagate not as far into the lateral dendrite as early APs because of recurrent inhibition (Egger and Urban, 2006). Third, asynchronous unitary release from GCs will broaden the response (Chen et al., 2000; Lage-Rupprecht et al., 2020; Ona Jodar et al., 2020). Lastly, activation of GCs beyond their threshold for lateral signaling requires more than one input to the GC, depends on the activity of both interconnected MCs, and might also occur in a delayed manner (Arevian et al., 2008; Giridhar and Urban, 2012; Burton and Urban, 2015; Lage-Rupprecht et al., 2020; Mueller and Egger, 2020). Thus, the functional or effective connectivity will vary dynamically.

The small size of unitary inputs and their substantial attenuation along the lateral dendrite make it unlikely that distal GC spine inputs activated *via* lateral excitation from other MCs will exert a substantial influence on somatic spike timing (see also McIntyre and Cleland, 2016), since these lateral inputs are unlikely to occur in similarly high numbers and temporal coincidence compared to recurrent inhibition. However, they can be expected to involve coordinated inhibitory inputs from GC glomerular ensembles associated with coactive columns (Egger and Kuner, 2021) and might shape recurrent output from the involved sets of GCs in a manner that indeed allows influencing MC temporal coding (McTavish et al., 2012).

Apparently, the overall structure of our connectivity estimate is reproduced fairly well by a reported measurement of lateral inhibition by Christie et al. (2001, their Figure 4). These data were obtained by recording IPSCs at MC and TC somata in acute brain slices in response to a glomerular stimulation electrode that was gradually stepped away from the glomerular region above the recorded cell. IPSC amplitude data were normalized to the IPSC amplitude in response to stimulation of the cell’s “home glomerulus.” This setting corresponds to the connectivity between a single MC and a glomerular ensemble (green lines in Figures 5C,D), even though a substantial amount of connections is removed within brain slices. *In vivo*, Peace et al. (BioRxiv 2017) used optogenetic glomerular activation, observing substantial lateral excitation of GCs even at 500 μm away and also far-ranging lateral inhibition of MCs. In other *in vivo* data sets, lateral inhibition was reported as strongest rather close to the home glomerulus and as occurring in a non-isotropic manner (Ezeh et al., 1993; Luo and Katz, 2001; Lehmann et al., 2016). In all such studies, however, the aforementioned EPL interneurons together with glomerular layer circuits are likely to dominate the recorded lateral MC inhibition because of the activity-dependence of GC-mediated lateral inhibition.

### Outlook

Previous findings indicate the existence of rather isolated functional glomerular column-like ensembles of GCs (Kauer and Cinelli, 1993; Willhite et al., 2006), which might form in an activity-dependent manner based on an isotropic substrate (Kim et al., 2011). Such ensembles may provide specific connectivity between populations of related MCs and TCs in different glomeruli. In our view, specific functional connectivity between coactive glomerular columns could emerge based on a coincidence detection mechanism that involves presynaptic NMDA receptors within the GC spine’s presynaptic active zone (Lage-Rupprecht et al., 2020); such connectivity might indeed be enhanced by learning, reconciling the existing divergent notions on patchy versus continuous olfactory mapping.

Since lateral inhibition is activity-dependent and thus unlikely to happen without recurrent inhibition, the main effect of lateral inhibition will be to amplify recurrent inhibition from the sets of GC spines belonging to mother GCs that are part of coactive glomerular columns. Therefore, these GCs fire global or dendritic spikes that also invade the spines (Egger and Kuner, 2021). How exactly this amplification is enacted will be the subject of future computational and experimental investigations. Certainly, it will increase the probability for GABA release, but by how much and during which time window after an MC AP? There are already experimental hints on the short-term plasticity of MC inputs to GCs (Dietz and Murthy, 2005; Chatterjee et al., 2016; Pressler and Strowbridge, 2017) and on the summation of Ca^2+^ entry into GC spines upon coincident local and global activation (Aghvami et al., 2019; Mueller and Egger, 2020). This question is also highly important in the context of repetitive MC firing during a theta-gamma burst – how are recurrent and possibly lateral release probability modified over time within a succession of MC spikes? Finally, how are the massive excitatory centrifugal cortical inputs onto GCs (e.g., Matsutani, 2010; Markopoulos et al., 2012) integrated with recurrent and lateral inhibition?

Extensions of our approach could be used to model TC subnetworks (Mori et al., 1983; Orona et al., 1983). In addition, the input from axon collaterals from MCs and various TC subtypes that are known to also terminate on GCs could also be accounted for. These types of inputs are highly likely to provide strong excitation and thus contribute to the formation of columnar GC ensembles (Pressler and Strowbridge, 2020); more detailed knowledge of these connections is required to estimate the number of GCs belonging to a glomerular ensemble (Figure 1A) that can be excited from a glomerulus beyond the (local) spiking threshold. Here, it would also be advisable to explore the effect of such inputs within the respective subnetworks of MCs and TCs, since their axonal collaterals within the bulb have different projection patterns (e.g., Kishi et al., 1984; Lodovichi et al., 2003; Hirata et al., 2019; Lukas et al., 2019; Sun et al., 2020).

Another interesting option might be to explore the impact of adult neurogenesis (Altman, 1969, recent review Tufo et al., 2022) on the bulbar network, depending on the percentage of exchanged GCs. Late newborn GCs are preferentially deep GCs (Lemasson et al., 2005) and may thus preferentially synapse onto MCs rather than TCs (Mori et al., 1983; Orona et al., 1983), influencing the MC subnetwork. We presume that the value of the synapse density on lateral dendrites n_LD_*MC*_ for juvenile rats observed here might increase later because of the overall doubling of GC numbers in rats in the course of development (Richard et al., 2010). Conversely, such an increase in contacts might be balanced by a reduction in release probability and other plastic adaptations.

## Data Availability Statement

The original contributions presented in this study are included in the article/supplementary material, further inquiries can be directed to the corresponding author.

## Ethics Statement

According to German animal ethics legislature, experiments in acute brain slices do not require approval by an ethics committee. Other rules apply (e.g., proof of qualification of the involved researchers for brain slice preparations has to be provided to the institutional veterinarians). For this study, brain slices were taken from ongoing experiments for Chatterjee et al. (2016).

## Author Contributions

VE conceived the study, constructed the anatomical network model, and wrote the manuscript. SA performed the functional simulations. YK contributed to the histochemical part of the study. All authors contributed to manuscript revision and read and approved the submitted version.

## Conflict of Interest

The authors declare that the research was conducted in the absence of any commercial or financial relationships that could be construed as a potential conflict of interest.

## Publisher’s Note

All claims expressed in this article are solely those of the authors and do not necessarily represent those of their affiliated organizations, or those of the publisher, the editors and the reviewers. Any product that may be evaluated in this article, or claim that may be made by its manufacturer, is not guaranteed or endorsed by the publisher.
